# The Gold Coast Integrated Care Programme: The Perspectives of Patients, Carers, General Practitioners and Healthcare Staff

**DOI:** 10.5334/ijic.5550

**Published:** 2021-05-07

**Authors:** Anne McMurray, Lauren Ward, Lei (Rachel) Yang, Martin Connor, Paul Scuffham

**Affiliations:** 1 School of Nursing and Midwifery, Menzies Health Institute Queensland, Griffith University, Queensland, AU; 2 Centre for Applied Health Economics, School of Medicine, Menzies Health Institute Queensland, Griffith University, Brisbane, AU; 3 Menzies Health Institute Queensland, Griffith University, Gold Coast, AU; 4 Menzies Health Institute Queensland, Griffith University, Queensland, AU

**Keywords:** integrated care, Australia, health services, primary care, multidisciplinary team, nurse navigator

## Abstract

**Introduction:**

The Australian Gold Coast Integrated Care programme trialled an innovative model of care to proactively manage high risk patients with complex and chronic conditions in collaboration with general practitioners. The objective was to enhance coordination and continuity of care across primary and secondary health services from a single point-of-entry multidisciplinary coordination centre. This case study, embedded in the broader trial, analysed the perceptions of patients, healthcare staff and general practitioners on the adequacy, comprehensiveness, timeliness and acceptability of the new model of care to help inform the decision by the health service whether to adopt it beyond the trial.

**Methods:**

This mixed method embedded, explanatory case study design included surveys of general practice staff and focus groups with patients, carers and coordination centre staff. Qualitative data were thematically analysed and findings merged with survey data in a narrative explanatory case report.

**Discussion:**

Staff, patients, general practitioners and practice nurses were generally satisfied with services, coordination of care and information sharing but general practice staff satisfaction ratings declined over time.

**Conclusion:**

The programme enhanced care and coordination of services and was valued by patients and healthcare providers. Study results provide a rationale for adopting the model for those with chronic and complex conditions.

## Introduction

The Australian Gold Coast Integrated Care (GCIC) programme was a four-year trial of an integrated model of care for patients with chronic and complex conditions. It was developed as a response to studies reporting that like other countries, Australia has a fragmented, inequitable and poorly coordinated healthcare system [1]. The trial was based on the hypothesis that a whole-system approach to providing care across primary and secondary care sectors would enhance health and wellbeing for vulnerable, high risk patients at no additional cost to the health system. The programme was conducted in a region with an older population that is rapidly growing at a greater rate than the rest of the country [2]. After community consultation with health service planners and administrators and local medical and health professionals the programme was designed and jointly funded by the Gold Coast Hospital and Health Service (GCHHS), the Gold Coast Primary Health Network (GCPHN), and Queensland Health. The objective was to work with patients to proactively manage their care in close collaboration with their general practitioners (GPs) and a multidisciplinary team of medical, nursing, pharmacy and allied health professionals located in a community Coordination Centre (CC). A pragmatic, mixed-methods non-randomised evaluation was supported by funding from the Australian Government Department of Health under the Workforce Redesign Programme, and conducted by the Centre for Applied Health Economics at Griffith University [3].

The aim of this case study, embedded in the broader evaluation, was to analyse the perceptions of patients, carers, general practice and healthcare staff on the adequacy, comprehensiveness, timeliness and acceptability of the clinical service delivery. These findings, together with the economic evaluation were intended to inform decisions on whether the model would be adopted beyond the trial period.

## Gold Coast Integrated Care: structures, processes and evaluation

The GCIC programme was guided by Valentijn et al.’s [4] conceptual framework; to operate on the macro (shared governance between care organisations), meso (disease status or subpopulation types), and micro level (identification of individual patient needs) [5]. To be congruent with international models of integrated care the GCIC programme ensured that all participants in the study were committed to patient-centred care [6,7,8,9]. Care organisations included the GCHHS, GPs from 15 local ‘network’ practices who had agreed to participate in the trial in response to a written invitation to all local practices, and a multidisciplinary team (MDT) of clinicians located in the CC. The CC team comprised two medical directors, medical specialists who visited on a rotating basis, and a group of 9–12 nurses and allied health practitioners (two occupational therapists, a pharmacist, social worker, psychologist, and physiotherapist). The team also included eight Nurse Navigators (NN) whose role was unique in being the first Australian NNs attached to general practices rather than a secondary or community health service. In addition to their role in general practice, each NN had a clinical role in the CC. They helped patients navigate health services by providing information and referrals from both locations as well as acting as a liaison between general practice staff and other health practitioners and services [10]. At the development stage the staffing complement consisted of six full time equivalent (FTE) clinical and six non-clinical staff, including two FTE research positions, with the number of clinicians varying throughout the programme as patient recruitment progressed. By the end of the trial there were 23 FTE clinical and 19 FTE non-clinical staff.

Patients were identified for the programme from hospital data and the recommendations of their GPs [3]. Information sharing was expedited by linking patient hospitalisation records with GP data, a system that was developed to facilitate the programme [11]. The programme evaluation recruited 1,549 patients from a potential of 7,400 patients with chronic conditions who had attended at least one of the 15 participating general practices. These were matched with 3,045 people (passive control group) in the catchment area who had previously accessed GCHHS hospital services, of whom 875 agreed to be part of an ‘active’ control group. These patients consented to releasing their hospital administrative data for the trial and provided patient reported outcome measures (PROM) as a basis for comparison with data from the GCIC study participants.

The provision of care was underpinned by an individualised holistic patient risk assessment. Their care plan was developed from this assessment and agreed to by the patient, GP and members of the MDT. Members of the MDT coordinated ambulatory care clinics, referrals and follow-up between the patient’s GP, medical specialists and other health service providers in the HHS, with home visits when necessary. Communication was enhanced through a Shared Care Record (SCR) which was purpose built for the programme. The SCR was accessible to patients, staff at the CC, practitioners from the general practices, and clinicians in the HHS. It housed a longitudinal clinical history and hospitalisation risk assessment, admission and discharge summary, appointment bookings, referral management, medication reports and a risk assessment and actions worksheet for each participant. Additional Information and Communication Technology (ICT) systems included disease registers. Servers for the registers were placed in all network general practices for data sharing between the practices and the HHS, and they included prevalence, distribution of disease information and key care management metrics for each practice. The disease registers were intended to enable prioritisation of interventions by benchmarking or ranking the clinical needs of patients according to their individual history and diagnostic group. Integration between the ICT and data strategies was achieved through a platform developed by in-house teams and co-designed with the clinical leaders of the programme. The ability to provide timely data to each element of the programme was enabled by an automated data matching process developed between general practice and hospital service data [11].

## Methods

### Study design

An embedded, explanatory case study was used to provide a descriptive analysis of the perspectives of patients, carers, general practice and CC staff on the adequacy, comprehensiveness, timeliness and acceptability of the programme. Case study is useful in asking ‘how’ and ‘why’ questions in a particular context bound by time, place and activity [12,13,14]. Embedding this case within the broader programme evaluation was intended to link the implementation of the programme with the programme effect (the economic analysis), as suggested by Yin (2017). The case reported here adopted a mixed method design. Qualitative data included analysis of focus groups conducted with patients, their carers and professional healthcare staff. Quantitative data include responses to the Assessment of Chronic Illness Care (ACIC) questionnaire (version 3) [15], and surveys of general practice staff practising as partners in the GCIC programme, including content analysis of open-ended comments. All authors of this paper were part of the evaluation team except MC, who was the programme leader and had oversight of programme planning and development activities.

### Data Collection

**Figure 1:**
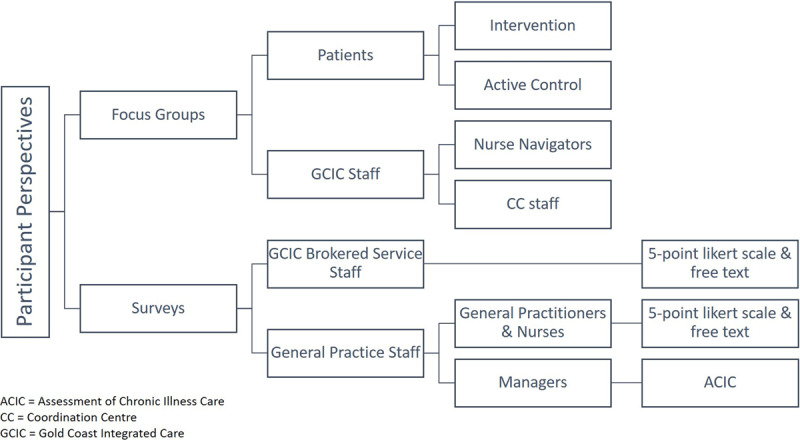
Data collection summary.

***Figure 1*** provides an overview of the data collected during the study.

#### Focus Groups with Patients and Carers

To recruit participants for the focus groups we used a computer-generated random numbers programme, Research Randomizer™ [16] to randomly select patients, until a quota of two from each practice was reached for each round of focus groups. Patients with dementia or a severe cognitive impairment were excluded. Patients and their carers were invited by phone to attend one of the focus groups held at a local venue and an interview protocol sent to those who agreed to attend. The one-hour focus groups were conducted 12 and 24 months after study recruitment commenced (2016 and 2017). The patients and their carers were prompted to discuss their overall experience and satisfaction with the programme, any comments on communication between their GP and the programme, coordination and timeliness of care, views on the holistic assessment, the SCR and any changes they had experienced since joining the programme.

At the second-year stage, a randomly selected group of patients and their carers who had been allocated to the active control group in the trial were invited by mail to attend one of four focus group sessions at a local community centre. Morning tea and refreshments were offered including an incentive to go in the draw to win a $100 gift card. These patients and their carers were prompted to discuss their health needs, communication with care providers and satisfaction with care and health providers.

#### Focus Groups with Staff Members

Focus groups with GCIC staff were conducted at three stages of the programme: seven groups in 2015; three groups in the final year (2018); and one group of NNs in 2016. Discussion prompts focused on their experience in developing the programme, reflections on working in the multidisciplinary team, any changes in their approach to patient care, their opinions on the communication between service providers, their professional development needs, and their commitment to the programme.

All focus groups were conducted by the first author (AM) with field notes recorded by the research assistant (LY) to ensure completeness of information. Focus groups were recorded and professionally transcribed. Data were thematically analysed by two researchers (AM, LW) using Braun and Clarke’s (2006) [17] analytic method and NVivo (version 11) software [18]. Both researchers reviewed the themes in conjunction with the field notes. Once themes from each group were identified, these were further reviewed for areas of similarity and data saturation.

#### General Practice Surveys

Annual purposively designed surveys were administered to all participating GPs and Practice Nurses (PNs) to canvass their opinions on the challenges and changes in providing care to chronic disease patients, and their satisfaction, expectations, communication and liaison with GCIC, GCHHS, and other services. Communication changes with patients and other health services were rated according to adequacy, comprehensiveness, effectiveness, accuracy, timeliness and satisfaction with specific components of the programme such as the NN role and ICT (Supporting text 1). Responses to all surveys were collected on a 5-point Likert scale with free text input. Surveys were completed online using Survey Monkey™ [19] or by hard copy provided by the practice NN. The surveys were not pilot tested as they were slightly modified to ask about changes as different aspects of the programme were introduced. Practice managers were also invited to complete the ACIC questionnaire (version 3) [15], an international validated survey evaluating the strengths and limitations in health care delivery when the practice was first enrolled in the programme (baseline) and on completion of the programme in 2018. The ACIC survey focuses on the six areas of the Chronic Care Model: organisation of the healthcare delivery system; community linkages; self-management support; decision support; delivery system design; and clinical information systems. Survey data were analysed in Microsoft Excel® using descriptive statistics including percentage frequency distribution and measures of central tendency (mean).

### Ethical approval

Ethical approval for the evaluation was received from the GCHHS (HREC/15/QGC/22) and Griffith University (MED/22/15/HREC). The trial is registered with the Australian New Zealand Clinical Trial Registry (ANZCTR) as a non-randomised controlled intervention study (Registration number 12616000821493).

## Findings

### Focus Groups

Focus group findings are referenced to the group rather than individuals to preserve the focus on group discussion and the anonymity of participants. Findings are coded as P1 or P2 for patients, C1 or C2 for carers depending on whether they report on groups in year one or year two. The active control group patients and carers are coded as ACP and ACC respectively. Staff focus group participants are coded as SFG 1, or SFG2, and Nurse Navigators as FGNN.

#### Patient and Carers’ Experience and Satisfaction

***Table 1*** presents the response rate for all patient and carer focus groups.

**Table 1:** Summary of patient focus group attendance.


FOCUS GROUP	TOTAL PATIENTS INVITED (N)	TOTAL PATIENTS ACCEPTED N (%)	TOTAL ATTENDEES			ATTENDEES GENDER	
	
			PATIENT N (%)	CARER (N)		MALE N (%)	FEMALE N (%)

*Intervention*							

Year 1 – 2016^a^	117	29 (25)	15 (52)	5		7 (35)	13 (65)

Year 2 – 2017^b^	139	58 (42)	33 (57)	2		23 (66)	12 (34)

*Active Control*							

2017^c^	400	63 (16)	45 (71)	10		22 (40)	33 (60)


^a^ Two focus groups; ^b^ Four focus groups; ^c^ Four focus groups.

Thematic analysis of these focus groups revealed the themes listed in ***Table 2*** below.

**Table 2:** Patient and carer focus group themes.


FOCUS GROUP	THEME

*Programme Patients*	

2016	• Information Sharing • Confusion about the role and function of GCIC • Satisfaction with extra services

2017	• Having improved care coordination • Closer engagement with health professionals • Trying to deal with unmet needs

*Active Control Patients*	

2017	• Satisfaction with GP • Appreciation for Health Services Received • Dissatisfaction with Hospital Waiting Times and Discharge Planning


GCIC = Gold Coast Integrated Care; GP = general practitioner.

A total of 15 programme participants and five carers attended the focus groups held in 2016, 12 months into the programme, most of whom (65%) were female. *Information sharing*, especially between GCIC and their GP was the most common theme, especially given the difficulties they had experienced with long waiting times in the Emergency Department and Outpatient Departments of the local hospitals.

*“I understand my medical records are accessible by the doctor and the hospital…it’s easier when I’m being checked into the hospital-they can go to my chart fairly instantly” (P1)*.

A second theme was *confusion about the role and function of GCIC*. Some were confused about the flow of information between the integrated care programme, the hospitals, and their GP, with some uncertainty in understanding the role of the SCR.

*“If you’re under a couple of different doctors at the hospital, does it just go into your record or is it under (named departments)? Maybe we should ask our GP” (P1)*.

A third theme *satisfaction with extra services* reflected their experiences in having the medication review and holistic assessment provided by GCIC as well as having accessed the telephone support provided by the MDT clinicians.

*“I have rung Integrated Care before I’ve spoken to mum’s doctor and it was satisfactory…lovely yes. I would definitely join the programme again” (C1)*.

At the second-year stage (2017), the patients and carers attended one of four focus groups at their convenience. These were attended by 33 programme participants and two carers, the majority of whom were male (66%). Interview prompts were similar to those of the 2016 focus groups, with the addition of a question on what had changed over the 12 months. At this stage participants were much more aware of the programme. Themes from this analysis included *having improved care coordination, closer engagement with health professionals and trying to deal with unmet needs*. The patients and their carers identified programme strengths as the personal phone calls to check on their health and progress, and the coordination of services in partnership with their GPs, which they found had improved timeliness, efficiency and effectiveness of the services they had accessed.

“*They followed up with a phone call the next day asking how it’s going [with] everything explained to me” (P2)*.

*“It’s like when [pharmacist] rang me up a lot of the times because of my medication and he was worried. Now he’s got it all sorted out” (P2)*.

Only one patient complained about the lack of responsiveness of the GCIC team, as he had received no follow-up. Others reported overall satisfaction with GCIC services for pain management, information and referrals to specialist care.

*“…trying to get an appointment with a specialist they can get in a lot quicker…you’re there to help me” (P2)*.

One patient commented that her GP was taking more of an interest in her, and another praised the relationship between her GP and the pre-admission clinic. Another was surprised that the NN and social worker contacted her during hospitalisation. However, some continued to have unmet needs in accessing home services from the Home and Community Care Programme and dental services.

*“Certain simple things like cleaning, gardening…but unless the government’s going to subsidise the people that go out and actually trim the hedge nobody’s going to do it” (C2)*.

#### Control Patients’ and Carers’ Experiences and Satisfaction

In 2017 the patients allocated as an active control group in the trial and their carers attended one of the focus groups at their convenience. A total of 45 patients and 10 carers attended a focus group; the majority (60%) of whom were females. Thematic analysis of these focus groups revealed three main themes: *Satisfaction with their GP, Appreciation for Services Received, and Dissatisfaction with Hospital Waiting Times and Discharge Planning*. Participants reported being generally well-informed about the health services they used. Patients were particularly enthusiastic about listening to others’ experiences and hearing of their self-management techniques in the context of the focus groups. Comments on their satisfaction with GPs focused on both clinical and interpersonal relationships.

*“My GP seems to be across everything. When I go in for my magical six minutes I have blood tests regularly. I have total confidence in him” (ACP)*.

*“The GP – he’s my brother, we talk family things…we can speak openly to one another as friends. If he sends you to a specialist and you’re not happy or don’t feel comfortable he says I’ll send you to someone until it’s right” (ACP)*.

Most had multiple experiences with the hospital, and they were generally supportive of local health services. One carer explained that the hospital is their care coordinator, establishing an emergency management plan and 24-hour telephone access for her husband’s care, however another found the waiting times for her sister’s specialist oncology care difficult.

*The care depends on how important the diagnosis is, “Heart problems and things like that…they’re straight onto it” (ACC)*.

The main complaints from this group were about needing better discharge information *“…trying to decipher what they’re saying [at discharge]”* including educational material to help them self-manage their conditions, and outpatient waiting times. Others expressed their understanding of the constraints on hospital personnel.

*“[the hospital has a 4.5 hour wait…the (OPD) system needs badly looking at…I think they’ve got the model completely wrong…they book five people at one time” (ACC)*.

*“I go to the public hospital. I can’t say enough in favour of them. To me, they’ve been marvellous” (ACP)*.

*“When you need to see a doctor they’re very good and they do the best they can, and tell you as much as they can tell you…it’s the waiting outside until you can get in” (ACP)*.

#### Staff Members’ Experiences and Perceptions

***Table 3*** outlines GCIC staff participation for the 2015 and 2018 focus groups.

**Table 3:** Summary of staff focus group attendance.


GCIC STAFF GROUP	2015 FOCUS GROUP	2018 FOCUS GROUP

Executive Management and Specialists	7	3

Service Navigators	4	4

Nurses	8	–

Nurse Navigators	–	5

Allied Health	3	3

Administration	4	2

ICT Team	7	3

Mixed group	4	0

Total Number of Attendees	37	20


GCIC = Gold Coast Integrated Care; ICT = information communication technology.

Thematic analysis of data from 2015 and 2018 focus groups is presented in ***Table 4***.

**Table 4:** Staff focus group themes.


FOCUS GROUP	THEME

2015	• Communicating across services • Embracing the work culture • Changing expectations • Transforming and adapting to change

2018	• Restructuring and refocusing • Redefining care processes • Articulating lessons learned


Seven focus groups were conducted in 2015, six months after the programme commenced patient enrolment, included with 37 clinical, technical (ICT) and administrative staff. Thematic analysis of the 2015 focus groups revealed that communication remained the priority, and team members explained its integral role in ensuring the programme remained patient-centred. However, they were challenged by the time pressures of communicating across services, especially with slow HHS services and wide variability among the general practices. Communication challenges also involved acquiring new ICT skills and developing “*strategy, systems, structures”* at the same time as they worked on recruiting patients.

*“We need to be “tech savvy” (SFG 1)*.

*“Engagement with the health care service is ‘undercooked’ with a long-standing challenge to connect the HHS and GPs (primary and secondary care systems)” (SFG1)*.

They described the organisational and work culture as having been influenced by strong leadership, with the goal of adapting and transforming work roles to deliver better quality care. Their commitment to the programme was unwavering as they responded to new situations.

*“I’ve done more varied work in the last four months than the previous four years, so it’s been a learning curve but allowed me to tap into things I was passionate about and interested in” (SFG1)*.

*“We’re moving from ‘my patch’ to what’s good for society (SFG 1)*.

Patient recruitment and assessment were lengthy processes, with considerable time required to gather data from older patients without undue burden. Once patients were recruited staff had to move between patient care and data management, including patient booking, scheduling assessments, initiation of the SCR, collection of evaluation data, and conducting liaison, referrals, care plan signoffs and other exchanges of information with GPs and the HHS. With programme expansion, staff worked closely with one another, building professional relationships, adapting to the variability in general practices, developing new linkages and strengthening existing ones.

*“We have to “knock on the door a few times” [to get some people to engage] (SFG 1)*.

Their expectations were shaped by the multidisciplinary team as they worked to develop and adapt protocols to ensure consistency in the information that was shared with hospital, general practice staff and the MDT clinicians.

*“You become a better professional by absorbing knowledge from other disciplines – for example, assessing the home environment while doing a medication review” (SFG1)*.

In 2018 as the clinical trial reached its final year, a further three focus groups were undertaken with 20 clinical, administrative and leadership participants. Thematic analysis revealed three themes: *Restructuring and Refocusing, Redefining Care Processes and Articulating Lessons Learned*. Participants discussed the programme in terms of the greater structural efficiency and effectiveness that had occurred.

*“[our] activities have become highly systematic” (SFG 2)*.

The changes they experienced included better valign="top" alignment of objectives and communication with patients, within the team, and with external stakeholders. They considered working in the MDT a strength that had a positive effect on care coordination.

*“Everybody is here because they believe in the mission, in what we are trying to achieve” (SFG2)*.

After three years they were able to articulate a number of aspects of the programme they would continue with or modify if it was to begin again. In the context of developing the holistic assessments, they had developed a useful risk management spreadsheet on patients who they determined as having avoided hospitalisation as a direct result of GCIC coordination. However, developing this type of tool also took considerable time away from clinical processes. Several members commented on their disappointment in the SCR, which was specifically developed for the programme. It had proven unwieldy and time consuming to develop and implement, with few general practices choosing to access patient data in this way. Staff members commented that the time taken to familiarise themselves and the practice staff on the SCR had taken up time they could have used more productively.

*“We shouldn’t have reinvented the wheel” (SFG 2)*.

*“Better to buy off the shelf and have value and the ability to tweak, rather than build something that would take years of development and testing” (SFG2)*.

They also believed the trial should have been longer and focused on a broader group of patients.

*”The paradigm shift to this type of programme should be an investment of 5–10 years to stop the next 11% of patients tipping into the 3%” (of high-risk patients) (SFG 2)*.

#### Nurse Navigators’ Experiences and Perceptions

Seven out of eight NNs participated in a NN specific focus group in 2016. Analysis revealed four overarching themes listed in ***Table 5***.

**Table 5:** Nurse Navigator focus group themes.


FOCUS GROUP	THEME

2016	• Enthusiasm and engagement with general practices • Challenging and variable workload • Maintaining a professional voice • Dealing with the ICT environment


ICT = information communication technology.

The NNs were enthusiastic about their new role, which was the first time NNs had been attached to general practices. They enjoyed getting to know the general practice staff as well as the patients, but they were also challenged by having to invent the role, especially with variability in general practices. An unexpected element of the role had been the amount of non-clinical work it involved. All group members agreed on the need to maintain their professional voice by having a nursing lead.

*“Our new roles are evolving. We all come from multiple backgrounds. We have different skills…it was just go forth and fix it” (FGNN)*.

*“We are all rolling it out a little different but on the same wavelength…every GP and every practice is different” (FGNN)*.

*“We’ve been advised that within the smaller teams the navigator is the central person. We’re a good team. We just don’t get to voice how we all feel together” (FGNN)*.

Most of the NNs had begun the programme working within the MDT. Once the NN role became more focused on their individual assignment to one or more general practices, some expressed concerns. The most challenging concern involved ensuring connectivity in the practices for the disease registers and overcoming technical difficulties with the SCR. Some of the NNs enjoyed working through the technology so they could assist practice staff; however, most could see the constraints on general practice from having to duplicate processes.

*“It’s building a picture of your patient” (FGNN)*.

*“I think a lot of these tools were developed without nursing input…without nursing understanding” (FGNN)*.

### General Practice Staff Perceptions

Response rates to the general practice surveys are presented in ***Table 6*** below. Findings presented are based on the following four key elements of the survey: *Satisfaction with the GCIC Programme, Communication with health services since joining the GCIC Programme, Communication with GCIC and other services*, and *Management of Chronic Care*.

**Table 6:** General Practitioner and Practice Nurse survey response rates.


SURVEY RESPONSE RATES	TOTAL INVITED N			TOTAL RESPONDENTS N (%)	
	
	GENERAL PRACTITIONER	PRACTICE NURSE		GENERAL PRACTITIONER	PRACTICE NURSE

Baseline (2015)	103	48		44 (43)	13 (27)

Year 1	109	35		55 (50)	19 (54)

Year 2	108	37		43 (40)	16 (43)

Year 3	96	35		49 (51)	6 (17)


#### Satisfaction with the GCIC Programme

**Figure 2:**
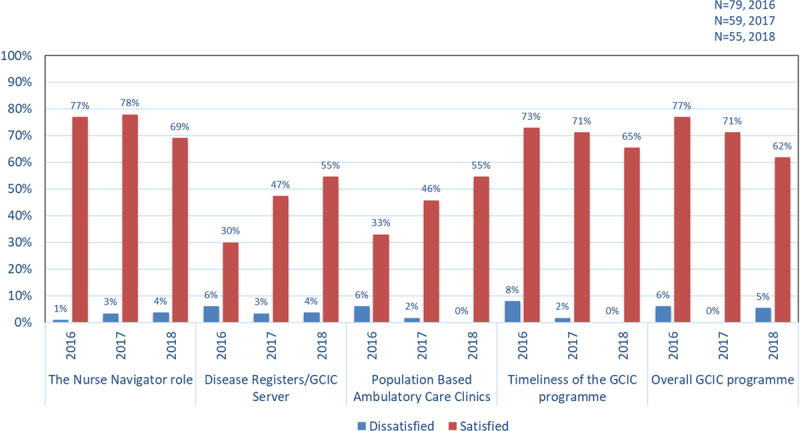
General Practitioner and Practice Nurse satisfaction with the programme (percentage of respondents).

Results showed that the majority of GPs and PNs were satisfied with all components of the programme, with a slight decline in satisfaction over time for all elements of the programme except Disease Registers/GCIC Server, and Ambulatory Care Clinics (***Figure 2***). At baseline the main issues were identified as managing chronic conditions, coordination and access to additional services, communication and patient compliance. In the final (2018) survey, most of the open-ended comments reflected recognition of the role of GCIC in enhancing patient engagement, sharing information and helping coordinate care plans. They also mentioned the expectation of their patients having fewer hospital presentations, improved patient services, and reduced service duplication. Programme strengths were identified as responsiveness, better patient identification, integrated assessment, care coordination and follow-up, including discharge information. The NNs were the most highly rated component of the programme, with the SCR the most difficult and unwieldy to navigate. They found the SCR added an additional burden to their workload because it required attending to an additional computer screen beyond their usual system for patient information as well as documenting this information in a new visual device that had been recently introduced by the HHS. This device, called ‘The Viewer’ [39], was introduced by Queensland Health to enable healthcare professionals, including GPs, quick access to patients’ information, including hospital discharge.

#### Communication with Health Services since Joining the GCIC Programme

**Figure 3:**
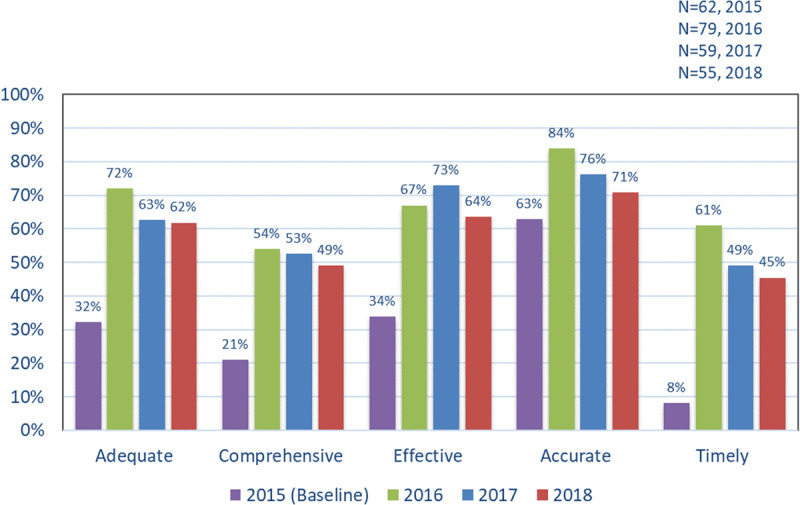
Proportion of general practice survey respondents who rated communication about their patients with GCHHS as mostly/always for each domain.

***Figure 3*** shows the proportion of practice staff rating communication about their patients with GCHHS at the ‘mostly or always’ level, in rating the programme for ‘adequate, comprehensiveness, effective, accurate and timely’. In 2016 communication was rated relatively high across the different domains, compared to baseline. However, 2017 and 2018 findings showed a gradual decline across all domains except ‘effectiveness’.

#### Communication with GCIC and Other Services

**Figure 4:**
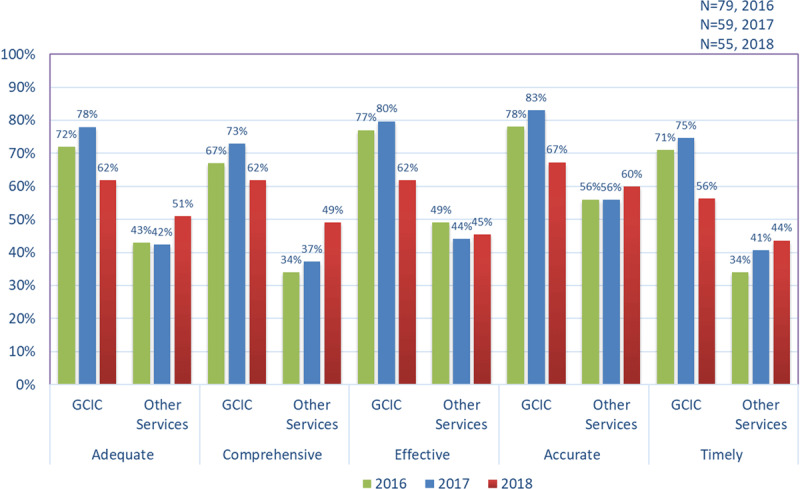
Proportion of general practice survey respondents who rated communication about their patients with GCIC and other services as mostly/always for each domain.

***Figure 4*** compares the proportion of survey respondents who selected the ‘mostly/always’ level of communication about their patients with GCIC and other services since joining GCIC. In both 2016 and 2018 communication with GCIC was rated significantly higher across the five domains ‘adequate, comprehensiveness, effective, accurate and timely’ than other services. However, these ratings also dropped in 2018. Respondents also judged both GCIC and other services as accurate, but with low ratings for timeliness since joining the GCIC.

#### Management of Chronic Care

**Figure 5:**
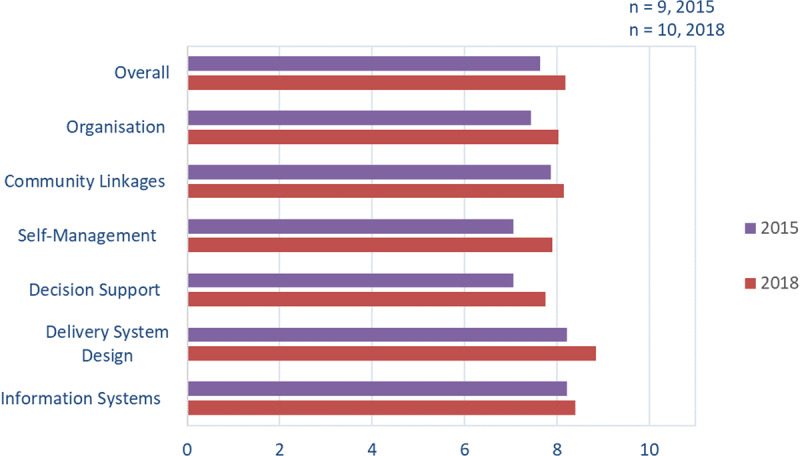
Average ACIC scores across domains.

Nine general practice managers returned the ACIC survey in 2015 (64%) and 10 in 2018 (67%). At baseline, all indicated that their practices had ‘*reasonably good support for chronic illness care*’, and one-third reported ‘*fully developed chronic illness care*’. By 2018, the proportion reporting ‘*fully developed chronic illness care*’ had increased to 60% (***Figure 5***).

## Discussion

Components of the GCIC programme were similar to other integrated care programmes described in the research literature [20]. The programme achieved its goal of providing comprehensive integrated care on a macro (shared governance between care organisations), meso (chronic disease status) and micro level (patient-centred care organised around individual needs) as intended in the conceptual framework [14]. Shared governance was evident in the organisational and professional integration between GPs and the multidisciplinary team of GCIC clinicians. Clinical integration was achieved from the holistic assessment and patient care plan developed with collaborative input from patients, their GP and GCIC clinicians. Functional integration was limited by the four-year project funding, but the generosity of funders was an indication of shared goals and culture, leadership and the integrated care vision [21]. Importantly the programme maintained its person-centred commitment as evident in the comments from patients, carers and staff. We were able to recruit the population sample we sought with careful consideration of the patient ‘voice’; an essential but under-researched element in many integrated care programmes [22].

Patients and carers, including those in the control group, were enthusiastic to be able to share their views in the focus groups. Their feedback on the programme resonates with the findings of a scoping review on older people’s perspectives of integrated care [23]. These researchers found that patients sought continuity, seamless transitions and good communication between services and settings with accessible coordinated care, self-care support, respect for their preferences and involvement of family members. Our patient-centred approach met these expectations in the context of the holistic approach to planning and care. Patient-centred care may not be exclusive to the GCIC programme, as feedback from control patients and their carers indicated the extent to which they felt well cared for by the Gold Coast GPs and the health services in general. An exception, as also reported in Lawless et al.’s study, was their concern about waiting times and the need for discharge information [23]. The GCIC patients were offered all their clinical information through the SCR, but like the GPs, they avoided using it even after being shown how to access their data. Their lack of interest in the technology was not surprising given the hesitancy of many older people to access electronic health data such as the Australian government’s *MyHealthRecord*. Despite being available since 2016, it is only recently that members of the general public have begun to access their health information [24].

The ongoing commitment and enthusiasm of GPs and their staff from the 15 network practices was a strength of the programme and most expressed a desire to see the programme continue beyond the trial. As with many primary care and public health collaborations the GCIC programme systems and processes were designed to foster strategic coordination and collaboration between the different levels of service [25]. Our primary care participants (GPs and practice staff) remained committed to the programme throughout its duration, providing what Valentijn et al. [4] describe as the integrative functions of primary care (first contact, continuous, comprehensive and coordinated). It is likely that the ACIC data showing improvements in chronic disease management over time may have been at least partly due to the collaboration between practices. However, we were surprised at the declining ratings of satisfaction and communication effectiveness in the third-year GP survey. The decline may be linked to the difficulties they experienced with the ICT systems, or the response burden of the survey. As others have suggested, it may be an indication that evaluating the complexity of integrated care programmes requires a shift of tactics to a more participatory form than traditional surveys or other methods [26]. It would have been ideal to bring GPs, patients, and health services staff together for a debriefing before the end of the trial but the logistics of this type of exercise was difficult for both patients with mobility issues and the GPs.

The positive communication between GPs and the CC staff reflected the importance of networks and teamwork as a mechanism for strengthening primary care [28]. The communication strategy built relationships that helped bridge the gap between primary and secondary service providers, which is a critical factor in successful integrated care programmes [25,29]. The data linkage between general practice and hospital systems was an important innovation as to date, there are few places in Australia where linked datasets are appropriate and available [30]. The experience of developing and implementing the first integrated care programme in the Gold Coast region was highly motivating for GCIC staff members. Staff member insights into the strengths and weaknesses of the programme will be invaluable for future planning. An important element identified was the need to have processes in place from the outset, which could have reduced recruitment time and extended the period of full implementation. Attention to the changing trajectory of patient needs is also addressed by Zulman et al. [31] who explain that patients may have high intensity needs in the initial stage while the clinicians are building trust and helping them modify health behaviours, followed by a subsequent reduction in health services utilisation during the latter stages. This supports the need for a longer period of full implementation. Developing a programme specific risk stratification tool was also time consuming. It would be preferable to seek standardised processes for risk stratification that could be used across different contexts, which is being considered by those developing the Australian trial of Health Care Homes [32].

Positioning the NNs in general practices was an enabling factor in bridging the gap between primary and acute care services, which has also been found in other studies [5]. Providing guidance and support for the ICT processes and enhanced assessment of patient and family needs across settings is integral to the navigator role [27,33]. International studies have found that using NNs effectively can reduce emergency presentations; for example, at Kaiser Permanente [34] and in Canadian integrated care systems [35]. Carter et al. (2018) [36] theorise that NNs can support integration at the micro level by working with individuals, families and providers on a shared plan of care. At the meso-level, they can promote capacity building among care providers, and at the macro-level there is the potential for system change by identifying needs and tailoring services accordingly. The GCIC NNs have been used as a model for an expanded role that is being implemented throughout the HHS. A similar role using nurse practitioners has been incorporated into an integrated care programme in another Queensland HHS to trial care transitions for older persons across acute, sub-acute and primary care [37].

### Limitations

A key limitation of the study is that general practice surveys were anonymous and therefore we were unable to examine individuals’ changes in perceptions over time or return our analysis for validation. The possibility of general practice staff turnover over the life of the programme played a key factor in the decision not to link survey respondents at each follow-up. We also recognise that the views expressed in the patient focus groups could be of those who are more engaged in their health, and therefore are not fully representative of the evaluation cohort.

### Lessons Learned

The weaknesses in the ICT systems, processes and structures was disappointing, especially when few integrated care programmes have fully shared electronic information management between patients and health providers [27,38]. The Viewer [39] introduced by Queensland Health also added to the duplication of information, and some GPs found it cumbersome to run two systems simultaneously. A programme in the Netherlands that developed an electronic information portal shared by patients and professionals also found that if this type of information platform creates extra work it can function as a barrier to patient care and decision-making [40].

As the trial ended, the programme concluded due to competing funding requirements. However, the programme continues to have an impact in several ways. First, the patients continue to have access to the telephone follow-up to support patient and family self-management for chronic conditions. Second, a decision was made by the GCHHS to address discharge planning as one of the most important issues identified by patients and GPs. A collaborative team that includes former GCIC staff members has subsequently developed a Complex Discharge Care (CDC) programme conducted from the GCHHS. CDC team members identify the necessary activities to enhance coordination of care for patients with complex care needs. These include patients requiring service assessment by various teams such as the Aged Care Assessment or Hospital in The Home team; those who may require subspecialty review or have home care needs such as equipment or transport; carer support needs; legal needs such as guardianship; interface with disability services; repatriation to an external facility; nursing home placement support; supported accommodation; or other forms of social, behavioural or community support. This initiative reflects an important outcome of the programme, especially as participants identified unmet needs for home services. A third impact has been continuation of the NN role with several of the NNs relocated to the hospital to provide service navigation, support, care and advice to specialist areas such as rehabilitation. All these innovations continue to improve the health of those with complex and chronic conditions.

As recommended in the OECD [28] report, high quality, accessible people-centred care requires the right resources, with the right organisation, and the right incentives. These include innovative payment models incentivising coordinated care for those with complex needs, with national and international efforts to measure their health outcomes. Evaluation of integrated care programmes is important to progress from temporary or project funding to structural funding, which requires evidence on programme effectiveness [40]. Then scaling up integrated care programmes as a sustainable innovation for a broader community will require local planning to ensure that network governance systems are embedded in the various communities in a way that is both person and community centred and takes into consideration the local ecological and social factors that add to system complexity [41,42]. This case study of GCIC patients and health care providers adds to the growing understanding of how integrated care is experienced in this context. Other researchers also maintain that focusing on the perceptions and experiences of those for whom integrated care programmes are designed can help redress the imbalance between theoretical knowledge of micro level strategies that are well-researched and the dearth of both theoretical and operational studies on implementation of meso and macro level strategies [6,21].

In summary, the most important lessons learned from the GCIC programme include the following:

- Information between patients, their GPs and other service providers is a crucial element in encouraging patient and family participation in care, especially for those with ongoing needs;
- The role of the GP is central to providing a collaborative, holistic and comprehensive model of care;
- The multidisciplinary team is a cornerstone of integrated care. Having the capability of a one-stop-shop such as the coordination centre facilitates essential services such as medication reviews, diagnostic tests, wound care, occupational therapies, patient and family counselling and appropriate referrals;
- Telephone guidance is highly regarded by patients as a lifeline to easing the burden of chronic illness, particularly in providing accurate health information and continuity of care; Telephone contact can also expedite patient response rates to evaluation; in this case, encouraging participation in the trial;
- The role of the NN provides an ideal bridge between primary and secondary care, promoting communication and continuity between service providers as well as patient information and guidance;
- Clinical programme management and administrative structures should be developed prior to implementation
- The introduction of new communication technologies should be accompanied by adequate and appropriate systems training. Where possible, modification of existing platforms can circumvent costly new innovations;
- Scaling up models of integrated care should be done with local and regional input and shared network governance to be sustainable.

## Conclusion

The programme has left a legacy in the Gold Coast community illustrating what can be achieved through collaborative care across general practice and hospital and health services along with many lessons learned. Given the expressed commitment from all levels of government to improving health and wellbeing for those with chronic and complex conditions we remain optimistic that in future, funding for integrated care will move from designated project support to broader state, territory and Commonwealth policies of providing comprehensive integrated care.

## Additional file

The additional file for this article can be found as follows:

10.5334/ijic.5550.s1 Supplementary File 1. General Practice Staff Survey.
