# Supplementary material for: The Gold Coast Integrated Care Programme: The Perspectives of Patients, Carers, General Practitioners and Healthcare Staff
Source: Int J Integr Care. 2021 May 7;21(2):18. doi: 10.5334/ijic.5550 (PMC8103853; doi:10.5334/ijic.5550)
Supplement: Supplementary File 1. — General Practice Staff Survey. [file ijic-21-2-5550-s1.pdf]

`

Supplementary File 1 - General Practice Staff Survey

Study ID:

Today's date (dd/mm/yyyy):____________________________

Name of General Practice you work at: _________________________________

INFORMATION ABOUT YOU

1. **Occupation (please tick one)**
   - ☐ General Practitioner
   - ☐ Practice Nurse

2. **Please indicate the length of time that you have been working in *this practice*:**
   - ☐ ≤ 1 year
   - ☐ 2-5 years
   - ☐ 6-10 years
   - ☐ 11-14 years
   - ☐ ≥ 15 years

3. **Which of the following categories best fits your age? (Please tick only one)**
   - ☐ 20 – 29 years
   - ☐ 30 - 39 Years
   - ☐ 40 - 49 Years
   - ☐ 50 - 59 years
   - ☐ 60 - 69 years
   - ☐ ≥ 70 years

4. **What is your *gender*?**
   - ☐ Male
   - ☐ Female

`

5. **a) Do you think that the use of MBS billable Chronic Disease Management items undertaken for chronic disease patients in your practice has changed since becoming part of GCIC?** *(GP Management Plans (721, 732), Team Care Arrangements (723), Multidisciplinary Care Plans (729) and Multidisciplinary Case Conferencing (735-758))*

- ☐ No
- ☐ Yes - Increased
- ☐ Yes - Decreased
- ☐ Not sure

b) Please indicate the factors that influenced your response to the previous question (tick more than one if necessary):

- ☐ Increased need for chronic disease management
- ☐ Chronic disease management no longer required
- ☐ Services coordinated by GCIC
- ☐ Increased time pressures
- ☐ Increased patient engagement
- ☐ Decreased patient engagement
- ☐ Increased practice focus on acute rather than chronic conditions
- ☐ Decreased practice focus on acute rather than chronic conditions

- ☐ Other____

c) Please comment____

____

____

`

6. Since joining the Integrated Care program, can you rate your communication about patients with *Gold Coast Hospital and Health Service* (Please tick one answer for each)

	Never	Rarely	Sometimes	Mostly	Always
**Adequate**<br>...the information I receive gives me an overview of patient care.	☐	☐	☐	☐	☐
**Comprehensive**<br>...the information I receive gives me a complete picture of patient's care.	☐	☐	☐	☐	☐
**Effective**<br>...the information enables me to continue care of patients.	☐	☐	☐	☐	☐
**Accurate**<br>...the information I receive is correct.	☐	☐	☐	☐	☐
**Timely**<br>...I receive information in time for it to be useful for patient's next surgery visit.	☐	☐	☐	☐	☐

7. Since joining the Integrated Care program, can you rate your communication about patients with *Gold Coast Integrated Care* (Please tick one answer for each)

	Never	Rarely	Sometimes	Mostly	Always
**Adequate**<br>...the information I receive gives me an overview of patient care.	☐	☐	☐	☐	☐
**Comprehensive**<br>...the information I receive gives me a complete picture of patient's care.	☐	☐	☐	☐	☐
**Effective**<br>...the information enables me to continue care of patients.	☐	☐	☐	☐	☐
**Accurate**<br>...the information I receive is correct.	☐	☐	☐	☐	☐
**Timely**<br>...I receive information in time for it to be useful for patient's next surgery visit.	☐	☐	☐	☐	☐

`

8. Since joining the Integrated Care program, can you rate your communication about patients with *other health services* (Please tick one answer for each)

	Never	Rarely	Sometimes	Mostly	Always
**Adequate** ...the information I receive gives me an overview of patient care.	☐	☐	☐	☐	☐
**Comprehensive** ...the information I receive gives me a complete picture of patient's care.	☐	☐	☐	☐	☐
**Effective** ...the information enables me to continue care of patients.	☐	☐	☐	☐	☐
**Accurate** ...the information I receive is correct.	☐	☐	☐	☐	☐
**Timely** ...I receive information in time for it to be useful for patient's next surgery visit.	☐	☐	☐	☐	☐

9. Please rate your overall satisfaction with the following:

	Very dissatisfied	Somewhat dissatisfied	Neither satisfied nor dissatisfied	Somewhat satisfied	Very satisfied
The Nurse Navigator role	☐	☐	☐	☐	☐
Disease Registers/GCIC Server	☐	☐	☐	☐	☐
Population Based Ambulatory Care Clinics	☐	☐	☐	☐	☐
Timeliness (or responsiveness) of the GCIC program in assisting with patient care	☐	☐	☐	☐	☐
Overall GCIC program	☐	☐	☐	☐	☐

a. **Please comment on any issues affecting your satisfaction with the aspects mentioned above:**

____

____

____

10. In your own words, what do you see as *the strengths* of the GCIC program?

`

11. **In your own words, can you identify any *limitations* of the GCIC program that could impact on your practice?**

Thank you for completing this survey.
Please return to your Practice Manager or GCIC Nurse Navigator for collection.
